# Analysis of Critical Current Dependence on Specimen Length and Crack Size Distribution in Cracked Superconductor

**DOI:** 10.3390/ma17010176

**Published:** 2023-12-28

**Authors:** Shojiro Ochiai, Hiroshi Okuda

**Affiliations:** 1 Elements Strategy Initiative for Structural Materials, Kyoto University, Sakyo-ku, Kyoto 606-8501, Japan; 2 Department of Materials Science and Engineering, Kyoto University, Sakyo-ku, Kyoto 606-8501, Japan; okuda.hiroshi.5a@kyoto-u.ac.jp

**Keywords:** new model analysis, cracking of superconducting layer, reduction of critical current, specimen length, largest crack size, size difference among cracks

## Abstract

In order to describe the dependence of critical current on specimen length and crack size distribution in the superconducting tape with cracks of different sizes, a Monte Carlo simulation and a model analysis were carried out, employing the model specimens of various lengths constituted of multiple short sections with a crack per each. The model analysis was carried out to evaluate the effects of the two factors on the critical current of a specimen. Factor 1 is the size of the largest crack in a specimen, and Factor 2 is the difference in crack size among all sections at the critical voltage of critical current. Factors 1 and 2 were monitored by the smallest ligament parameter among all sections constituting the specimen and by the number of sections equivalent to the section containing the largest crack at the critical voltage of the critical current of the specimen, respectively. The research using the monitoring method revealed quantitatively that the critical current-reducing effect with increasing specimen length is caused by the increase in the size of the largest crack (Factor 1), and also, the critical current-raising effect is caused by the increase in the difference of crack size (Factor 2). As the effect of Factor 1 is larger than that of Factor 2, the critical current decreases with increasing specimen length. With the present approach, the critical current reducing and raising effects under various crack size distributions were evaluated quantitatively as a function of specimen length, and the specimen length-dependence of critical current obtained by the Monte Carlo simulation was described well.

## 1. Introduction

Critical current *I*_c_ (estimated by application of the electrical field criterion *E*_c_ = 1 µV/cm to the voltage V—current *I* curve) and *n*-value (estimated as the index *n* of the approximated *I* ∝ *I^n^* curve in the electrical field range of *E* = 0.1~10 µV/cm) are reduced under high electromagnetic and mechanic stresses, as have been reported for the superconducting oxide (such as RE(Y, Sm, Dy, Gd,···)Ba_2_Cu_3_O_7-δ_: REBCCO) layer-coated tape [1,2,3,4,5,6,7,8,9,10,11,12,13,14], and Bi2223 [15,16,17,18,19,20]-, MgB_2_ [21,22,23,24,25]- and Nb_3_Sn [26,27,28,29,30,31]-filamentary types. In this work, we conduct a fundamental study on the influences of cracks in superconducting layer-coated tape on *I*_c_. Usually, the cracking takes place non-uniformly, and hence, the *I*_c_/*n*-values vary by specimen [3,7,13,18] and along the length of the specimen [7,13,14]. Not limited to the stress-induced cracks, the non-uniformly distributed defects introduced during the fabrication process also cause a reduction in *I*_c_ and *n*-values [11,13]. For safety design, it is required to reveal the relation of non-uniformly distributed defects/cracks to superconducting property [32].

In studying the effect of crack size distribution and specimen length on *I*_c_ and *n*-values, the authors used a Monte Carlo simulation method [13,14] combined with a current shunting model of cracks [17]. From the simulation results using the model specimens consisting of multiple short sections with cracks of different sizes, the following results have been obtained: (i) The section with the largest crack contributes most significantly to the synthesis of the *V*–*I* curve of the specimen. (ii) The *I*_c_-value of a specimen is determined by the size of the largest crack in the specimen as a first approximation when the specimen is short but not when the specimen is long [9]. The size of the largest crack determines the *I*_c_ value of the specimen as a first approximation when the specimen is short but not when the specimen is long [13]. This result suggests that the *I*_c_ value is determined not only by the effect of the largest crack but also by an effect whose contribution to *I*_c_ becomes greater the longer the specimen.

Motivated by the results above, we attempted to describe the specimen length-dependence of the *I*_c_ value based on the effects of two factors. Factor 1 is the size of the largest crack in the specimen. Factor 2 is the difference in size among cracks at the critical voltage of the critical current of the specimen. As the research tools, we used the monitoring method for numerical estimation of the effect of Factor 1 and Factor 2, together with the Monte Carlo simulation method [13,14] and Gumbel’s extreme value distribution function [33].

## 2. Materials and Methods

### 2.1. Simulation to Obtain Critical Current Values under Various Specimen Lengths and Various Distribution Widths of Crack Size

**Figure 1:**
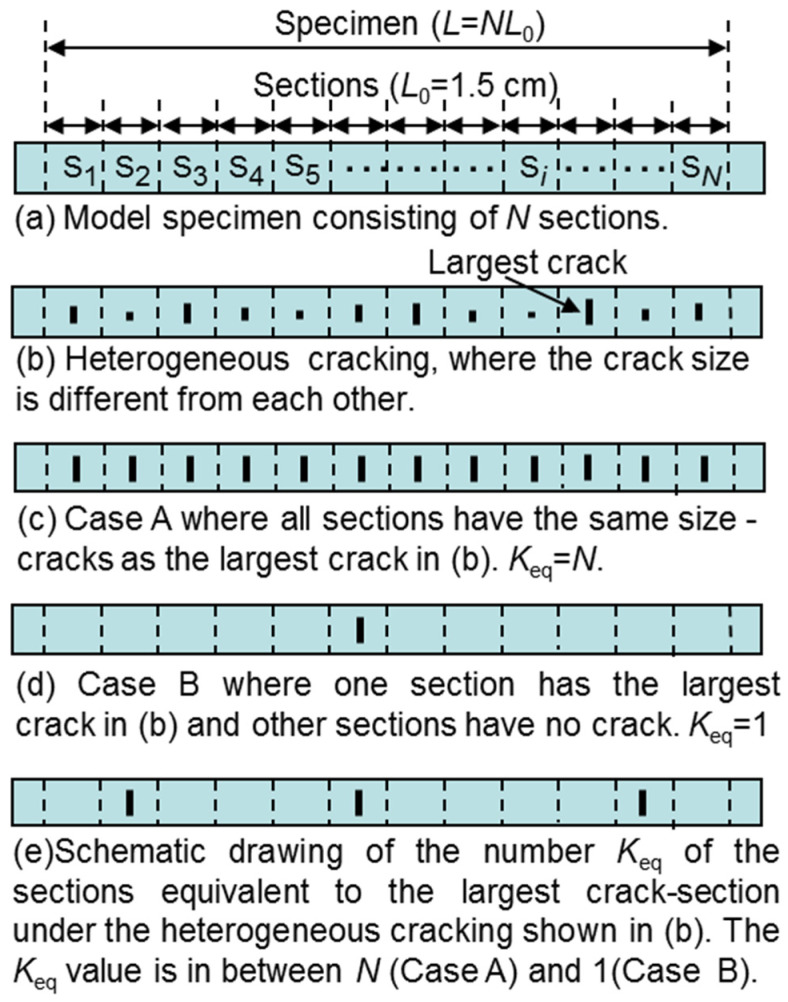
Schematic drawing of the model specimen and array of cracks for analysis. (**a**) Model specimen consisting of *N* local sections that have a length *L*_0_ = 1.5 cm and one crack in each. (**b**) Heterogeneous cracking in the model specimen. (**c**,**d**) The extreme Case A and Case B, giving the lower- and upper- bound of critical current, respectively. (**e**) Drawing of an example of the number of the sections equivalent to the largest crack-section Keq estimated from the heterogeneous cracking shown in (**b**) by the procedure shown later in Section 2.2. The *K*eq-value is in between N (Case A) and 1 (Case B). The case of *K*_eq_ = 3 is drawn as an example. The specimen length (voltage probe distance) *L* = *NL*_0_ length *L* = 1.5~60 cm under various crack size distributions were obtained by Monte Carlo simulation [13,14] combined with the crack-induced current shunting model [17].

Figure 1 shows (a) a model specimen of a superconducting layer such as REBCCO-layer-coated tape, containing *N* local sections with a length *L*_0_ = 1.5 cm and one crack per each, (b) heterogeneously cracked superconducting layer, (c) extreme Case A, (d) extreme Case B and (e) array of the largest crack in the intermediate case between Cases A and B, which were used as the tools to estimate the effects of Factor 1 and Factor 2 on *I*_c_. as follows:

The simulation was carried out in the following procedure. Then, the simulation results were analyzed to clarify the effect of Factor 1 (size of the largest crack) and Factor 2 (difference of crack size at the critical voltage of critical current of the specimen) on the *I*_c_ of the specimen by applying the method and procedure in Section 2.2.

The following technical terms were used. “*f* and 1 − *f*: ratios of cross-sectional area of the cracked- and ligament-parts to the total cross-sectional area of the superconducting layer, respectively”, “*I*_RE_: current transported by the superconducting layer in the ligament part”, “*I*_s_: crack-induced shunting current”, “*R*_t_: electrical resistance of the shunting circuit”, “*V*_RE_: voltage developed at the ligament part that transports current *I*_RE_, being equal to the voltage vs. (=*I*_s_*R*_t_) developed at the cracked part by shunting current *I*_s_, since the cracked part and the ligament part constitute a parallel electrical circuit”, “*I*_c0_ and *n*_0_: critical current and *n*-value of the sections in the non-cracked state, respectively”, “*E*_c_ (=1 μV/cm): critical electrical field for determination of the critical current value”, “*s* (<<*L*_0_): current transfer length”, “*L*_p_: ligament parameter of section, given by *L*_p_ = (1 − *f*)(*L*_0_/*s*)^1/*n*0^, which was derived by the authors [13,14] through a modification of the formulations of Fang et al. [17]”, and “Δ*L*_p_: standard deviation of the ligament parameter *L*_p_”.

The cross-sectional area of ligament 1 − *f* and crack *f* have a one-to-one relation; the smaller the ligament, the larger the crack. The ligament parameter *L*_p,_ which is proportional to 1 − *f*, was used as a monitor of 1 − *f* and *f*. Accordingly, the size of the largest crack is expressed by the smallest ligament parameter *L*_p,smallest_. The standard deviation of 1 − *f* and *f* is the same. Thus, the standard deviation Δ*L*_p_ of the ligament area fraction 1 − *f* is the same as that of the crack area fraction *f*. Hence, the standard deviation of the ligament parameter, Δ*L*_p_, was used as a monitor of the distribution width of crack size and ligament size. As well as that of crack size, the larger the Δ*L*_p_ value, the wider the distribution of both the crack size- and ligament size.

The distribution of *L*_p_ was formulated by the normal distribution function as an example. Noting the average of *L*_p_ values as *L*_p,ave_, the cumulative probability *F*(*L*_p_) and density probability *f*(*L*_p_) are expressed by:

(1) $$F(L_{p})=\frac{1}{2}\left\{1+\mathrm{erf}\left(\frac{L_{p}-L_{p,\mathrm{ave}}}{\sqrt{2}\Delta L_{p}}\right)\right\}$$

(2) $$f(L_{p})=\frac{1}{\sqrt{2\pi }\Delta L_{p}}\exp\left\{-\frac{(L_{p}-L_{p,\mathrm{ave}}){}^{2}}{2(\Delta L_{p}){}^{2}}\right\}$$

In the Monte Carlo simulation, the *L*_p_ value for each section was set by the following process. The *L*_p,ave_ was taken to be 0.667, which refers to a situation where the average crack size is ≈1/3. The standard deviation of *L*_p_, Δ*L*_p_ was set to be 0.01, 0.025, 0.05, 0.10, and 0.15. Using the values mentioned above, the *L*_p_ value was given for each cracked section by generating a random value in the range of 0~1, setting *F*(*L*_p_) = generated random value, and substituting the values of *L*_p,ave_ (=0.667) and Δ*L*_p_ (0.01–0.15) in Equation (1).

For the section without crack, the voltage (*I*)—current (*I*) relation is expressed by:

(3) $$V=E_{c}L_{0}\left(\frac{I}{I_{c0}}\right){}^{n_{0}}$$

The transport current in a cracked section is the sum (*I* = *I*_s_ + *I*_RE_) of the shunting current *I*_s_ in the cracked part and superconducting layer (for instance, REBCO)-transported current *I*_RE_ in the ligament part. The *V*–*I* curve of the cracked section is expressed as:

(4) $$V=E_{c}L_{0}\left(\frac{I}{I_{c0}}\right){}^{n_{0}}+V_{\mathrm{RE}}$$

(5) $$I=I_{\mathrm{RE}}+I_{s}=I_{c0}L_{p}\left[\frac{V_{\mathrm{RE}}}{E_{c}L_{0}}\right]{}^{1/n_{0}}+\frac{V_{\mathrm{RE}}}{R_{t}}$$

We calculated the *V*–*I* curve of the non-cracked section by substituting *L*_0_ = 1.5 cm, *I*_c0_ = 200 A, and *n*_0_ = 40 taken from the experimental result of DyBCO coated conductor [3], into Equation (3), and also *V*–*I* curve of the cracked section by substituting the *L*_p_ value given in the Monte Carlo method and the values mentioned above into Equations (4) and (5).

Since the specimen consists of a series electrical circuit of *N* sections (Figure 1a,b), the current *I* in the specimen is the same as that in all sections (Equation (6)), and the voltage of the specimen is the sum of the voltages of all sections (Equation (7)).

*I* = *I*_S_*_i_* (*i* = 1 to *N*) (6)

(7) $$V=\sum_{i=1}^{N}V_{S_{i}}$$

The *V*–*I* curves of the specimens were synthesized with the *V*–*I* curves of the sections using Equations (6) and (7). Using the *V*–*I* curves of the sections and specimens, the *I*_c_ values of the sections and specimens were obtained using the electrical field criterion of *E*_c_ = 1 μV/cm.

### 2.2. Model Analysis of the Specimen Length (L)-Dependence of Critical Current (I_c_) under Various Distribution-Widths of Crack Size (ΔL_p_)

A series of sections with cracks of different sizes (Figure 1b) constructs a specimen.

With increasing applied current, the superconductivity of the specimen is lost first in the section with the largest crack since the voltage developed at the largest crack section is highest among all sections, and hence, it contributes most to the voltage of the specimen. The ligament parameter of the largest crack section is the smallest among all sections. It is noted as *L*_p,smallest_. Factor 1 (largest crack in the specimen) was monitored by *L*_p,smallest_. When the *L*_p,smallest_ value is known, the lower and upper bounds of critical current can be calculated using the extreme Case A and Case B [13,14], as follows.

Case A is an extreme case where the crack size is the same as the largest of all sections, as shown in Figure 1c, and the *V*–*I* curves of all sections are the same. Accordingly, the voltage of the specimen, given by the sum of the crack size of the voltages of all sections, corresponds to the upper bound of the voltage of the specimen, *V*_upper_. As *V*_upper_ reaches *V*_c_ at the lowest current, the *I*_c_ value in Case A is the lower bound, *I*_c,lower_.

Case B is another extreme case where the crack size of one section is far larger than that of the other sections, as shown in Figure 1d, and the voltages developed at the cracks in the other sections are too low to contribute to the voltage of the specimen. This case gives the lower bound of the voltage of specimen *V*_lower_. As *V*_lower_ reaches *V*_c_ at the highest current, the *I*_c_ value in Case B is the higher bound, *I*_c,upper_, under the given largest crack size, monitored by *L*_p,smallest_.

In this way, the *V*_upper_–*I* curve and *I*_c,lower_ are given by Case A, and the *V*_lower_–*I* curve and *I*_c,upper_ are given by Case B due to the difference in the number of the largest cracks; *N* in Case A and 1 in Case B.

As shown in Figure 1b, cracks of various sizes exist in practical specimens. Accordingly, the largest crack section and the other sections contribute to the voltage of the specimen. The extent of the contribution of the other sections is seen in the positional relation among the *V*–*I* curves of the sections, reflecting the distribution-width of crack size Δ*L*_p_; when Δ*L*_p_ is small, the *V*–*I* curves of sections exist near each other and hence the voltage of the specimen (=sum of the voltages of sections) tends to be high, while, when Δ*L*_p_ is large, the *V*–*I* curves of sections exist apart to each other and hence voltage of the specimen tends to be low. This means that the difference in the size of cracks is an important factor in determining the *I*_c_ value.

The effect of the difference in size among cracks (Factor 2) on the *I*_c_ value of the specimen can be obtained by expressing the sum of the voltages of all sections (=the voltage of the specimen) as the sum of the voltages of several *K*_eq_ sections equivalent to the largest crack-section [14] and *N*–*K*_eq_ sections without cracks. A large *K*_eq_ value corresponds to a small difference in crack size. In this direction, Factor 2 (size difference among cracks) is monitored by *K*eq; the smaller the size difference of cracks, the larger the *K*eq. The *K*_eq_ value can be estimated as follows.

Using the smallest ligament parameter *L*_p,smallest,_ the *V*–*I* curve of the largest crack section is expressed by Equations (8) and (9), which are the modified forms of Equations (4) and (5), respectively.

(8) $$V\left(\mathrm{smallest}\;\mathrm{ligament}\text{-}\mathrm{section}\right)=E_{c}L_{0}\left(\frac{I}{I_{c0}}\right){}^{n_{0}}+V_{\mathrm{RE}}$$

(9) $$I=I_{\mathrm{RE}}+I_{s}=I_{c0}L_{p,\;\mathrm{smallest}}\left[\frac{V_{\mathrm{RE}}}{E_{c}L_{0}}\right]{}^{1/n_{0}}+\frac{V_{\mathrm{RE}}}{R_{t}}$$

The *V*–*I* curve of the specimen, containing a number of *K*_eq_ sections equivalent to the largest crack (smallest ligament)-section and the number of *N* − *K*_eq_ sections without cracks, having original critical current *I*_c0_, is expressed by:

(10) $$V(\mathrm{specimen})=E_{c}L\left(\frac{I}{I_{c0}}\right){}^{n_{0}}+K_{\mathrm{eq}}V_{\mathrm{RE}}$$

The *K*_eq_ value is between 1 and *N*, as shown in Figure 1c–e.

**Figure 2:**
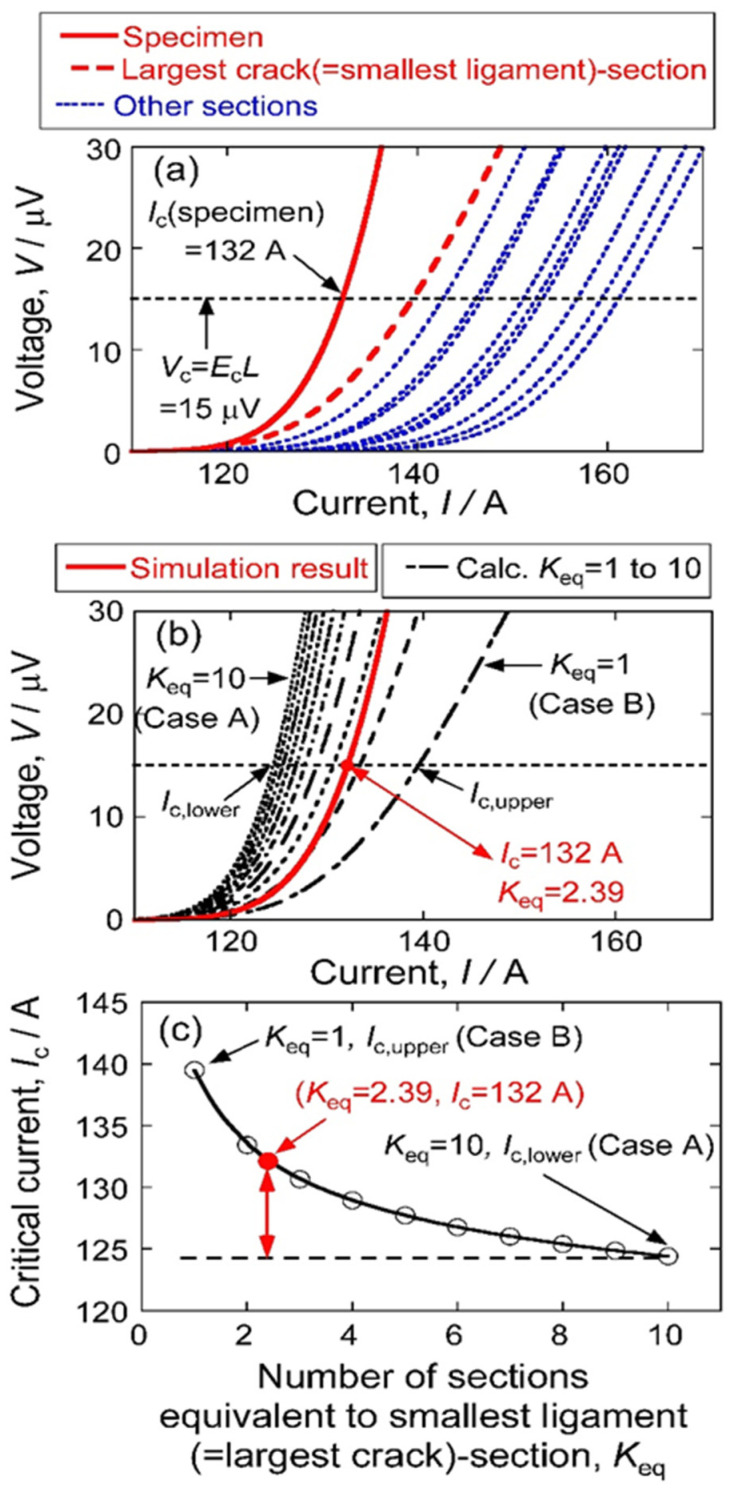
Relation of the critical current *I*_c_ to the number of sections equivalent to the smallest ligament (largest crack) section, *K*_eq_, where data of the 15 cm specimen taken from the simulation result are used as an example. In this example specimen, *L*_p,smallest_ was 0.617, and *I*_c_ was 132 A. (**a**) *V*–*I* curves of the example specimen (solid curve), the smallest ligament section (broken curve), and the other sections (dotted curves). (**b**) Comparison of the *V*–*I* curve of the example specimen (solid curve) with the *V*–*I* curves calculated with *K*_eq_ = 1 to 10. The *K*_eq_ value, corresponding to *I*_c_ = 132 A for *L*_p,smallest_ = 0.617 of the example specimen, was estimated to be 2.39. (**c**) The relation of the *I*_c_ value to *K*_eq_ value for *L*_p,smallest_ = 0.617, shows the increase in *I*_c_ value from the lower bound (Case A) to the upper bound (Case B) with decreasing *K*_eq_ from 10 to 1. The location of *I*_c_ = 132 A at *K*_eq_ = 2.39 in the *I*_c_ − *K*_eq_ diagram of the example specimen is shown with the closed circle.

Figure 2 shows the relation of *K*_eq_ to *I*_c_ and the procedure to estimate the *K*_eq_ value from the estimated values of *I*_c_ and *L*_p,smallest_ values. Figure 2a shows the *V*–*I* curves of the specimen with 10 sections (solid curve), and *V*–*I* curve of the largest crack section (broken curve), and the *V*–*I* curves of the other 9 sections whose cracks are smaller than the largest crack (dotted curves), obtained in advance by the present work. *L*_p,smallest_, was 0.617, and *I*_c_ was 132 A in this example.

Figure 2b compares the *V*–*I* curve of the specimen obtained by simulation and the *V*–*I* curves of the specimen calculated with *L*_p,smallest_ = 0.617 for *K*_eq_ = 1 to 10. At *V* = *V*_c_ = 15 μV, the *I*_c_ value of the specimen obtained by simulation was 132 A. This value is between the *I*_c_ values calculated with *K*_eq_ = 2 and 3. It is noted that not only the integers but also the decimal places can be used as the *K*_eq_ value for the calculation of the *V*–*I* curve and *I*_c_ of the specimen since Equations (8)–(10) hold mathematically for positive real numbers, including decimal places. Using Equations (8)–(10), the *K*_eq_ value that describes the simulation result of *I*_c_ = 132 A at *V* = *V*_c_ = 15 μV for *L*_p,smallest_ = 0.617 was 2.39, as shown in Figure 2b.

Figure 2c shows the relation of the *I*_c_ value to *K*_eq_ value for *L*_p,smallest_ = 0.617 in 15 cm-specimen, obtained from the *V*–*I* curves for *K*_eq_ = 1 to 10 in Figure 2b. It is shown that the lower bound of critical current *I*_c,lower_ corresponding to *K*_eq_ = 10 in this example, is determined by the effect of Factor 1 (size of the largest crack, which is monitored by *L*_p,smallest_) and the effect of Factor 2 (size difference among cracks, which is monitored by *K*_eq_) plays a role in raising *I*_c_ from *I*_c,lower_. In this way, the effects of Factor 1 and Factor 2 on *I*_c_ could be evaluated separately.

## 3. Results

**Figure 3:**
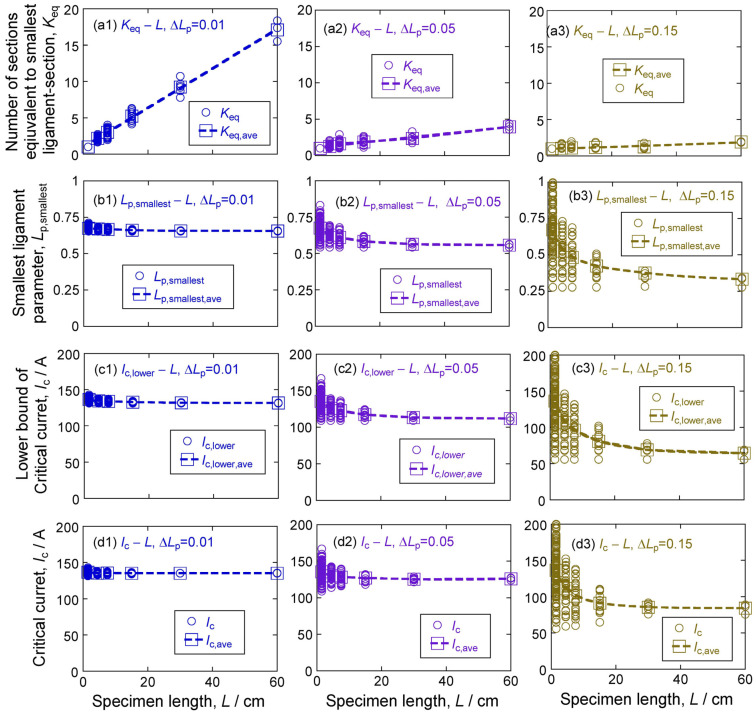
Plots of number of sections equivalent to the smallest ligament section *K*_eq_ (**a1**–**a3**), smallest ligament parameter *L*_p,smallest_ (**b1**–**b3**), lower bound of critical current *I*_c,lower_ (**c1**–**c3**) and critical current *I*_c_ (**d1**–**d3**), against specimen length *L*. These data were obtained by simulation and analysis for the standard deviation of the ligament parameter Δ*L*_p_ = 0.01 (**a1**–**d1**), 0.05 (**a2**–**d2**), and 0.15 (**a3**–**d3**). The value of each specimen is shown with a circle ○, and the average value under the combination pair of *L* value and Δ*L*_p_ value is shown with a square □.

The number of sections equivalent to the smallest ligament section *K*_eq_, smallest ligament parameter among all sections *L*_p,smallest_, lower bound of critical current *I*_c,lower_ and critical current *I*_c_ with an increase in specimen length *L* (1.5~60 cm) were investigated for a wide standard deviation of ligament parameter values Δ*L*_p_ = 0.01 to 0.15. Figure 3 shows the plots of *K*_eq_, *L*_p,smallest_, *I*_c,lower_, and *I*_c_ against *L*, obtained for Δ*L*_p_ = 0.01, 0.05, and 0.15. The *K*_eq_ values were obtained by analysis of the simulation results with the approach in Section 2.2. The *L*_p,smallest_, *I*_c,lower_, and *I*_c_ values were obtained by simulation. The value of each specimen is shown with a circle (○) and the average value with a square (□). While the *K*_eq_, *L*_p,smallest_, *I*_c,lower_, and *I*_c_ values are different from specimen to specimen, the average values (*K*_eq,ave_, *L*_p,smallest,ave_, *I*_c,lower,ave_, and *I*_c,ave_) show their correlation to *L* and Δ*L*_p_.

**Figure 4:**
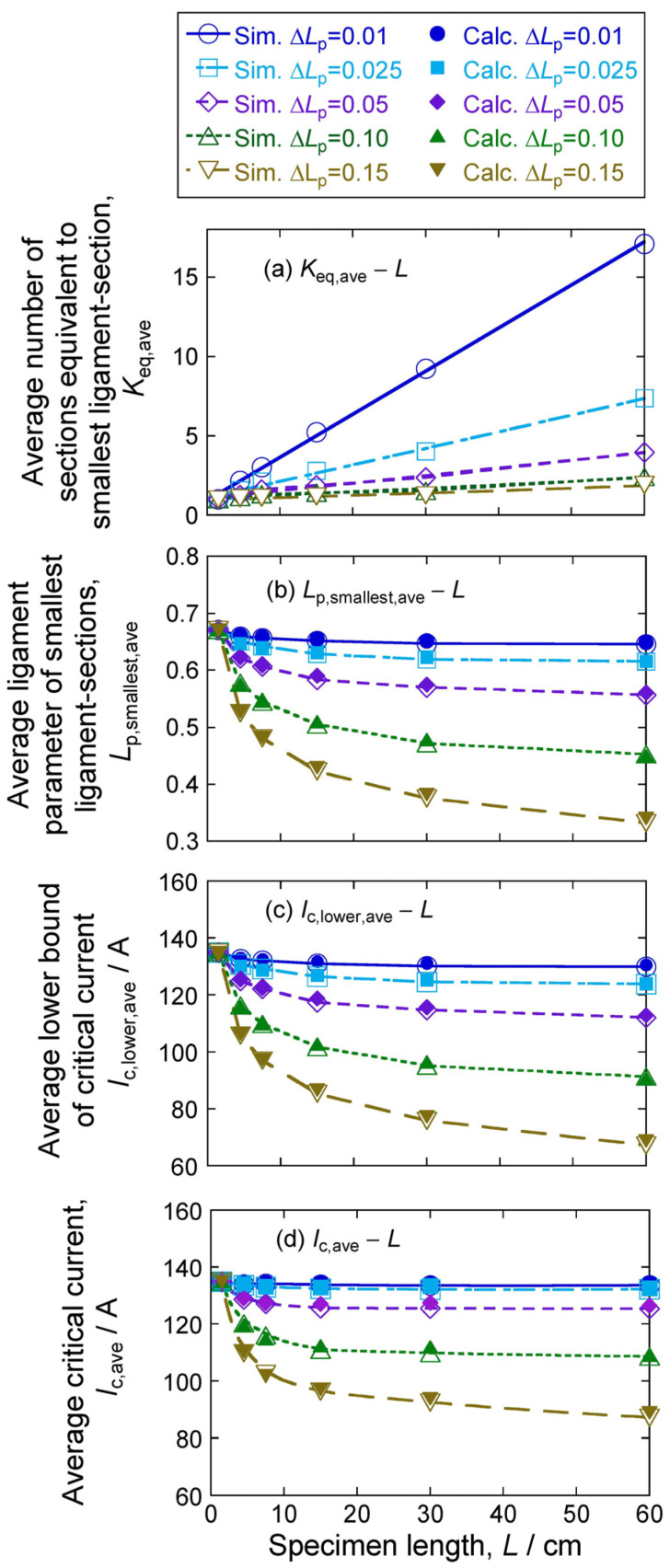
Changes of the average values of (**a**) *K*_eq,ave_, (**b**) *L*_p,smallest,ave,_ (**c**) *I*_c,lower,ave_, and (**d**) *I*_c,ave_, with an increase in specimen length *L* under the indicated value of Δ*L*_p_. The open and closed symbols show the values obtained by simulation and calculation, respectively.

Figure 4 shows the plots of an average of (a) number of sections equivalent to the largest crack section, *K*_eq,ave_, (b) smallest ligament parameter, *L*_p,smallest,ave_, (c) lower bound of critical current, *I*_c,lower,ave_, and (d) critical current, *I*_c,ave,_ with an increase in *L* in each case of Δ*L*_p_ = 0.01, 0.025, 0.05, 0.1, and 0.15. The open symbols for *K*_eq,ave_, *L*_p,smallest,ave_, *I*_c,lower,ave_, and *I*_c,ave_, show the results obtained by simulation. The closed symbols in Figure 4b–d show the *L*_p,smallest,ave_, *I*_c,lower,ave_, and *I*_c,ave_ values obtained by calculation, whose method is shown in Section 4.1.

The following features are read in Figure 4:

1. The average smallest ligament parameter *L*_p,smallest,ave_ decreases with an increase in *L*. Namely, the average size of the largest crack increases with an increase in *L*. The extent of the increment of the size of the largest crack with *L* is enhanced with an increase in Δ*L*_p_;
2. Average critical current *I*_c,ave_ decreases with an increase in specimen length *L*. The extent of the decrease in *I*_c,ave_ with *L* increases with the increase in the standard deviation of the ligament size (=standard deviation of crack size) Δ*L*_p_;
3. The change in the average of critical current *I*_c,ave_ and the average of the lower bound of critical) current *I*_c,lower,ave_ with an increase in specimen length *L* is similar to the change in *L*_p,smallest,ave_. This result suggests that the decrease in *L*_p,smallest,ave_, namely the increase in the size of the largest crack, has a significant influence on the determination of critical current.

The features mentioned above will be analyzed numerically from the viewpoint of the effects of Factor 1 and Factor 2 on *I*_c_.

## 4. Discussion

### 4.1. Calculation of Average Values of the Smallest Ligament Parameter, Lower Bound of Critical Current, Critical Current, and the Correlation between the Smallest Ligament Parameter and Lower Bound of Critical Current

Gumbel’s extreme value distribution function [33] describes the distribution of the extreme values of the smallest ligament (largest crack) parameter *L*_p,smallest_ in the present specimens, and the calculation results are compared with the present simulation and analyzed results. The cumulative probability of *L*_p,smallest_ value, ϕ (*L*_p,smallest_), and the average *L*_p,smallest_ value, *L*_p,smallest,ave_, for each set of Δ*L*_p_ and *L* values under the given value of *L*_p,ave_ (=0.667 in this work) are expressed as Equations (11)–(14).

(11) $$\Phi (L_{p,\mathrm{smallest}})=1-\exp\left\{-\exp\left(\frac{L_{p,\mathrm{smallest}}-\lambda }{\alpha }\right)\right\}$$

*L*_p,smallest,ave_ = λ − αγ (12)

λ and α are the positional and scale parameters, respectively, and γ is the Euler–Mascheroni constant (=0.5772). The values of λ and α under given values of *L* and Δ*L*_p_ are obtained by using Equation (1) (cumulative distribution function of *L*_p_: *F*(*L*_p_)) and Equation (2) probability density function *L*_p_: *f*(*L*_p_)) and the following relational expressions:

*F*(λ) = 1/*N* (13)

α = 1/{*N f*(λ)} (14)

We can calculate *L*_p,smallest,ave_ for given values of *L* and Δ*L*_p_ with Equations (1), (2), (12)–(14). Then, we can calculate the *V*_upper,ave_–*I* curve (Case A), which gives the average lower bound for critical current using Equations (9) and (10). From the calculated *V*_upper,ave_–*I* curve, we can obtain the *I*_c,lower,ave_ value for a given combination pair of *L* value and Δ*L*_p_ value with the criterion of E_c_ = 1 μV/cm. By substituting *L*_p,smallest_ = *L*_p,smallest,ave_ in Equation (9) and *K*_eq_ value (Figure 4a) in Equation (10) and applying the E_c_ = 1 μV/cm criterion, we can calculate the *I*_c,ave_ values for *L* = 1.5 to 60 cm and Δ*L*_p_ = 0.01 to 0.15.

The calculation results (closed symbols) of the average values of *L*_p,smallest,ave_: smallest ligament parameter, *I*_c,ave_: critical current, and *I*_c,lower,ave_: lower bound of critical current in comparison with the simulation results (open symbols) are shown in Figure 4b–d.

The simulation results of *L*_p,smallest,ave_, *I*_c,lower,ave_, and *I*_c,ave_ values were described well by calculation. With the estimated valuers above, the effects of Factor 1 and Factor 2 on the specimen length-dependence of critical current can be estimated separately, as shown below.

### 4.2. Separate Assessment of Effects of Factor 1 and Factor 2 on Specimen Length-Dependence of Critical Current

**Figure 5:**
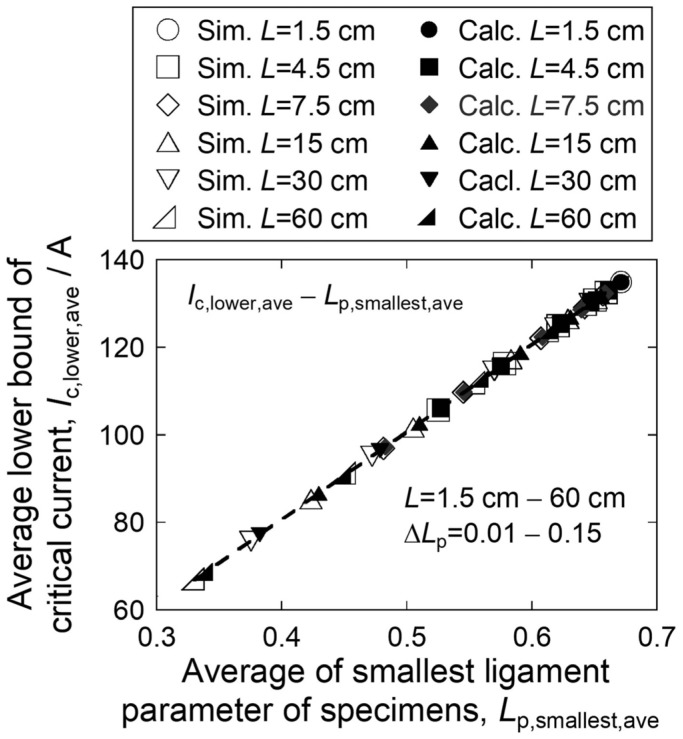
Plots of average values of the lower bound of critical current *I*_c,lower,ave_ against average values of smallest ligament parameter *L*_p,smallest,ave_. The data were taken from the results in Figure 4b,c. The values obtained by simulation and calculation are shown with the open and closed symbols, respectively.

**Figure 6:**
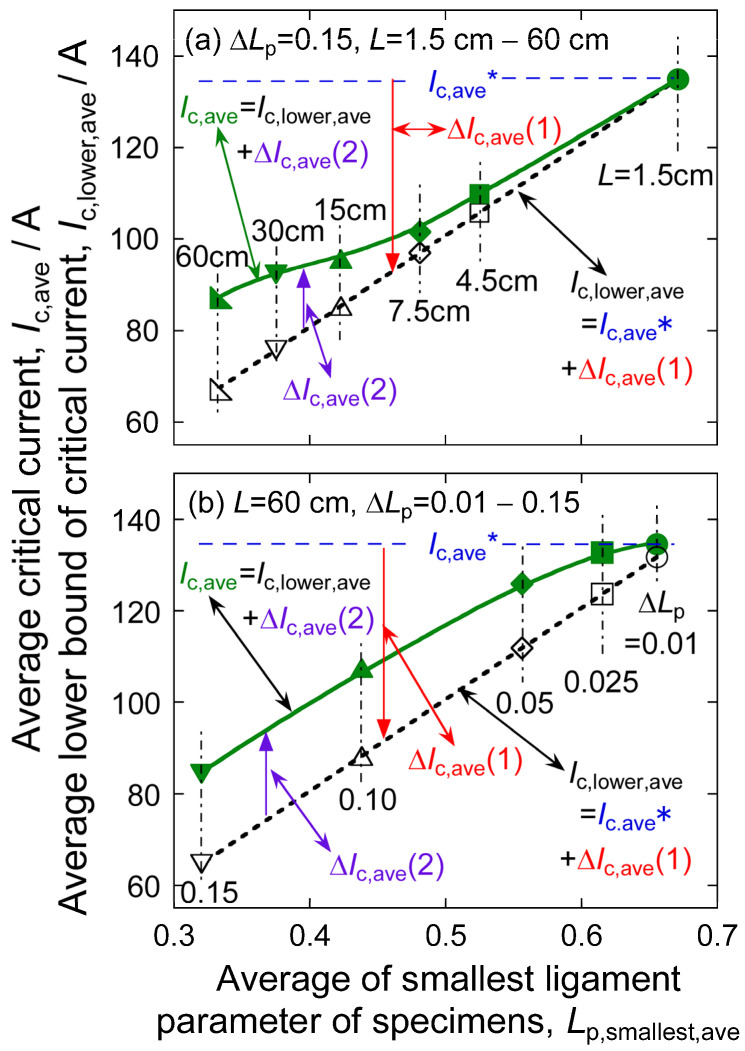
Plots of average critical current *I*_c,ave_ and average lower bound of critical current *I*_c,lower,ave_, obtained by simulation under the condition of (**a**) standard deviation of the ligament parameter Δ*L*_p_ = 0.15, and specimen length *L* = 1.5 cm to 60 cm, and (**b**) Δ*L*_p_ = 0.01 to 0.15 and *L* = 60 cm, against the average smallest ligament parameter *L*_p,smallest,ave_. The relations of *I*_c,ave_ to *L*_p,smallest,ave_ and *I*_c,lower,ave_ to *L*_p,smallest,ave_ are presented with the solid and broken lines, respectively. The average critical current of the specimens in the case of Δ*L*_p_ = 0 is noted as *I*_c,ave_* (=135 A in this work). This value is kept for any specimen length. The change of critical current Δ*I*_c,ave_ (1) caused by the increase in Factor 1 and the change of critical current Δ*I*_c,ave_ (2) caused by an increase in Factor 2 are indicated with arrows.

The changes of the average critical current *I*_c,ave_ of specimens with increasing specimen length were described with Factor 1 monitored by *L*_p,smallest,ave_ and Factor 2 monitored by *K*_eq,ave_, as shown in Figure 4b. It is noted that, as in Figure 4c, the *I*_c,lower,ave_ values of the simulation results were well described by calculation using Case A in which all sections have the same *L*_p,smallest,ave_ value (*K*_eq_ = *N*). In this case, Factor 1 is zero, and hence, this effect does not arise. Namely, the *I*_c,lower,ave_ value reflects just Factor 1. Plotting the *I*_c,lower,ave_ values in Figure 4c against the corresponding *L*_p,smallest,ave_ values in Figure 4b, we have Figure 5. It is clearly shown that the *I*_c,lower,ave_ has almost one to one relation to the *L*_p,smallest,ave_ for any specimen length *L* and any crack size difference Δ*L*_p_. Using this feature, the effects of Factor 1 and Factor 2 on the specimen length-dependence of critical current can be assessed separately in the following manner:

Figure 6 shows the plots of *I*_c,ave_ and *I*_c,lower,ave_, obtained by simulation under the condition of (a) Δ*L*_p_ = 0.15 and *L* = 1.5 cm to 60 cm, and (b) Δ*L*_p_ = 0.01 to 0.15 and *L* = 60 cm, against *L*_p,smallest,ave_. The solid and broken lines show the relations of *I*_c,ave_ to *L*_p,smallest,ave_ and *I*_c,lower,ave_ to *L*_p,smallest,ave_, respectively. The average critical current of the sections for any length is noted as *I*_c,ave_* (=135 A). The value of *I*_c,ave_* = 135 A is equal to the critical current value that appears if uniform cracking takes place under the condition of Δ*L*_p_ = 0 and *L*_p,ave_ = 0.667. This value is kept for any specimen length under Δ*L*_p_ = 0. The change of critical current Δ*I*_c,ave_ (1) caused by the increase in Factor 1 and the change of critical current Δ*I*_c,ave_ (2) caused by the increase in Factor 2 at the critical voltage with increasing *L* and Δ*L*_p_ are indicated with arrows.

Evidently, with a decreasing average of the smallest ligament parameter *L*_p,smallest,ave_, namely with increasing size of largest crack, values of *I*_c,ave_ and *I*_c,lower,ave_ decrease, Δ*I*_c,ave_ (1) increases more in a minus direction caused by decreased *I*_c,lower,ave,_ and Δ*I*_c,ave_ (2) increases caused by an increase of the difference between *I*_c,ave_ and *I*_c,lower,ave_. In this way, an increase in specimen length *L* and an increase in width of crack size distribution Δ*L*_p_ lead to an increase in Factor 1, which reduces critical current, and an increase in Factor 2, which raises the critical current by Δ*I*_c,ave_ (2).

**Figure 7:**
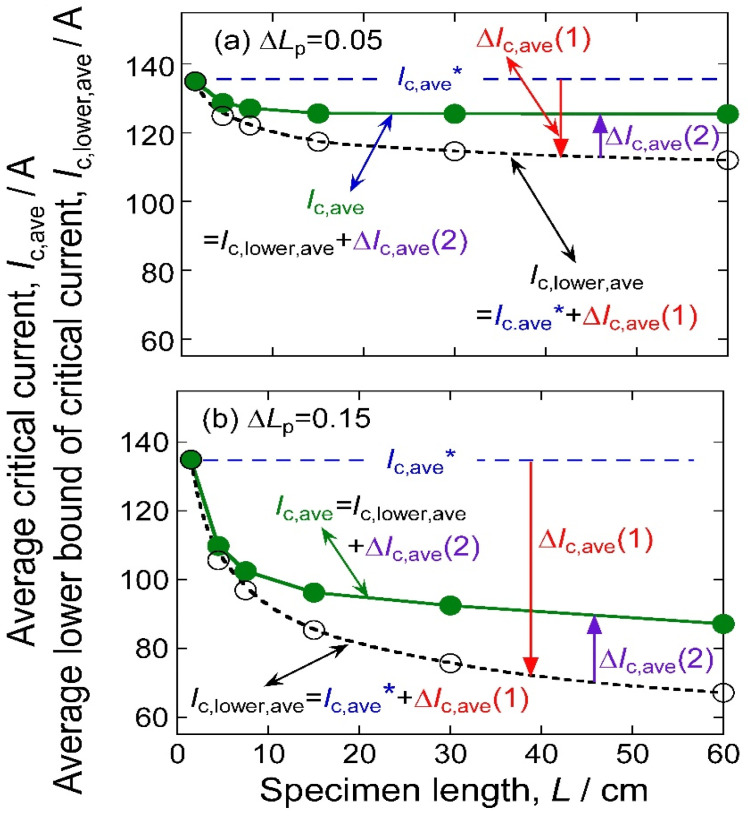
Changes in average values of critical current *I*_c,ave_ and lower bound of critical current *I*_c,lower,ave_ with increasing specimen length *L* in the cases of the standard deviation of ligament parameter Δ*L*_p_ = (**a**) 0.05 and (**b**) 0.15. Δ*I*_c,ave_ (1) (=*I*_c,lower,ave_ − *I*_c,ave_*) refers to the critical current-reducing effect arising from the increase in Factor 1 (size of the largest crack). Δ*I*_c,ave_ (2) (=*I*_c,ave_ − *I*_c,lower,ave_) refers to the critical current-raising effect arising from an increase in Factor 2 (difference in size among cracks).

Figure 7 shows *I*_c,ave_, *I*_c,lower,ave_, Δ*I*_c,ave_ (1) and Δ*I*_c,ave_ (2) plotted against specimen length *L* for Δ*L*_p_ = (a) 0.05 and (b) 0.15. As stated above, if cracking occurs uniformly (Δ*L*_p_ = 0) as the geometry of Case A (*K*_eq_ = *N*), the critical current value does not vary with specimen length, and it is kept to be *I*_c,ave_* (=135 A). In practical specimens, cracking occurs heterogeneously (Δ*L*_p_ > 0), and hence, the critical current value decreases from the *I*_c,ave_* value with increasing specimen length *L* and width of crack size distribution Δ*L*_p_.

As shown in Figure 7, with increasing *L*, the *I*_c,ave_ is reduced from *I*_c,ave_* to *I*_c,lower,ave_ by Δ*I*_c,ave_ (1) caused by the effect of increase in Factor 1. The critical current *I*_c,ave_ is given by the sum of the *I*_c,lower,ave_, and effect of Factor 2, Δ*I*_c,ave_ (2), as shown in Figure 2c, Figure 6 and Figure 7. The relations of Δ*I*_c,ave_ (1) and Δ*I*_c,ave_ (2) to *I*_c,ave_*, *I*_c,lower,ave_, and *I*_c,ave_, shown in Figure 6 and Figure 7, are expressed below.

The size of the largest crack is different from specimen to specimen. The average size of the largest crack in non-uniform cracking is monitored by *L*_p,smallest,ave_ in the present approach, which can be calculated by Equations (12)–(14). Setting *L*_p,smallest_ to be equal to the calculated *L*_p,smallest,ave_ value and *K*_eq_ = *N* in Equations (8)–(10), we can calculate *I*_c,lower,ave_ value. We can calculate the *I*_c,ave_ values by substituting *L*_p,smallest_ = *L*_p,smallest,ave,_ and *K*_eq,ave_ values shown in Figure 4a in Equations (8)–(10). From the value of *I*_c,ave_* = 135 A and the simulated and calculated *I*_c,lower,ave_ and *I*_c,ave_ values as a function of *L* for each Δ*L*_p_ value (0.01~0.15), the Δ*I*_c,ave_ (1) and Δ*I*_c,ave_ (2) can be estimated by Equations (15) and (16) from the simulation result and also from the calculation result.

Δ*I*_c,ave_ (1) = *I*_c,lower,ave_ − *I*_c,ave_* (15)

Δ*I*_c,ave_ (2) = *I*_c,ave_ − *I*_c,lower,ave_ (16)

**Figure 8:**
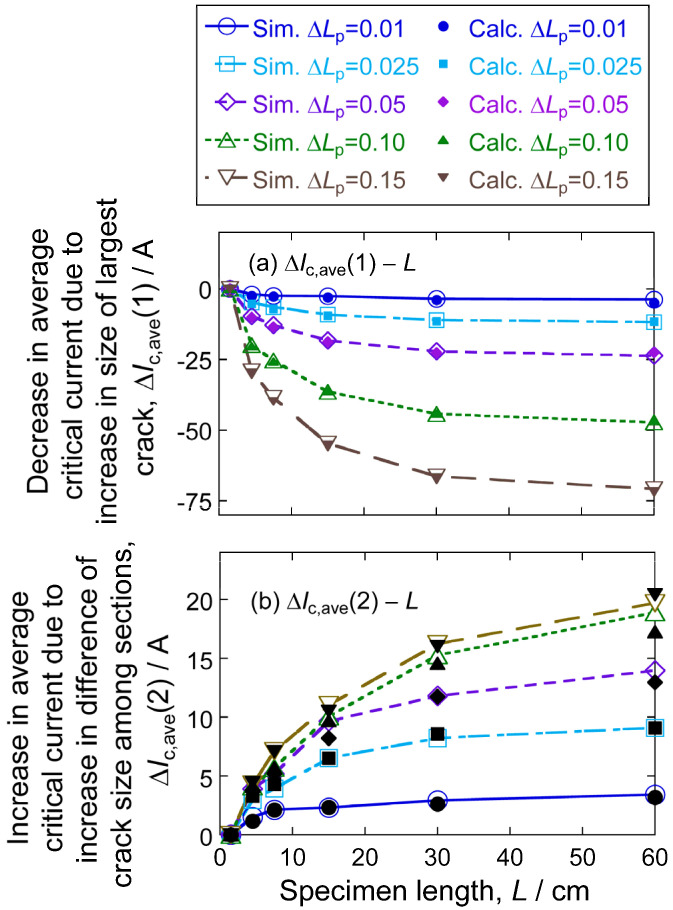
Estimated specimen length-dependence of the change in average critical current (**a**) caused by the change of Factor 1, Δ*I*_c,ave_ (1), and (**b**) caused by the change of Factor 1, Δ*I*_c,ave_ (2). The results of simulation and calculation are shown with open and closed symbols, respectively.

Figure 8 shows specimen length dependence (a) Δ*I*_c,ave_ (1) and (b) Δ*I*_c,ave_ (2) for Δ*L*_p_ = 0.01 to 0.15, estimated by simulation (open symbols) and by calculation (closed symbols). The following features are read:

(1) Δ*I*_c,ave_ (1), arising from the increase in the size of the largest crack (Factor 1), increases in minus direction with increasing specimen length *L* and standard deviation of crack-size distribution Δ*L*_p_;
(2) Δ*I*_c,ave_ (2), arising from the difference in crack size (Factor 2), increases in the plus direction with increasing *L* and Δ*L*_p_;
(3) From Figure 7, the effect of *I*_c,ave_ (1) and Δ*I*_c,ave_ (2) on *I*_c,ave_ with increasing specimen length;
(4) The change in the minus (Δ*I*_c,ave_ (1))—and plus (Δ*I*_c,ave_ (2))—effects with specimen length becomes moderate in long specimens.

*L* is expressed as

*I*_c,ave_ = *I*_c,lower,ave_ + Δ*I*_c,ave_ (2) = *I*_c,ave_* + Δ*I*_c,ave_ (1) + Δ*I*_c,ave_ (2) (17)

In this work, *I*_c,ave_* is 135 A for any *L*. Accordingly, whether *I*_c,ave_ increases or decreases with increasing *L* depends on the value of Δ*I*_c,ave_ (1) + Δ*I*_c,ave_ (2). Figure 8a,b demonstrates that the *I*_c,ave_ decreases with increasing specimen length *L* since the minus effect Δ*I*_c,ave_ (1) arising from Factor 1 is larger than the plus effect Δ*I*_c,ave_ (2) arising from Factor 2.

## 5. Conclusions

Using a model analysis and a Monte Carlo simulation method, critical current dependence on specimen length was studied by monitoring the effects of Factor 1: size of the largest crack, and Factor 2: size difference among cracks in cracked superconductor. The following results were obtained.

(1) The calculation- and simulation-results showed the following features of the specimen length-dependence of the critical current. (a) Large Factor 1 plays a role in reducing critical current. (b) Large Factor 2 plays a role in raising critical current under a given size of the largest crack. (c) The effect of Factor 1 is larger than that of Factor 2, and hence, the critical current decreases with increasing specimen length. This feature is enhanced with increasing standard deviation of crack size;
(2) For a quantitative description of the results mentioned in (1), the effect of Factor 1 on the critical current of the specimen was formulated by combining the smallest ligament parameter, corresponding to the largest crack section, with a shunting of the current model at the crack. The effect of Factor 2 on critical current was formulated using the number of sections equivalent to the largest crack section at the critical voltage of the specimen’s critical current. With the application of the present approach to the calculation and simulation results, the following results were obtained: (a) The critical current-reducing effect caused by an increase in Factor 1 and the critical current-raising effect caused by the increase in Factor 2 were assessed separately, and the critical current of the specimen was described as a function of specimen length. (b) The features mentioned in (1) were quantitatively described.

Present methods of simulation and calculation are simple, and reliable data are obtained. Thus, the methods and the results in this work are expected to be used for analysis and confirmation of experimental data concerning the relation between critical current, specimen length, and crack size distribution, which provides useful design information to ensure safety and reliability in the practical applications of superconductors.

## Data Availability

The data presented in this study are available on request from the corresponding author.
